# Long-term outcomes of an acellular dermal matrix for the treatment of complex cryptoglandular anal fistula: a pilot study

**DOI:** 10.1007/s10151-022-02593-1

**Published:** 2022-02-25

**Authors:** M. J. Gómez-Jurado, M. Martí-Gallostra, G. Pellino, A. Galvez, E. Kreisler, S. Biondo, E. Espín-Basany

**Affiliations:** 1 Department of Advanced Medical and Surgical Sciences, Colorectal Surgery, Vall d’Hebron University Hospital, Barcelona, Spain; 2 Department of Advanced Medical and Surgical Sciences, Università Degli Studi Della Campania “Luigi Vanvitelli”, Naples, Italy; 3 Colorectal Unit, Department of General and Digestive Surgery, Bellvitge University Hospital, University of Barcelona, and IDIBELL (Bellvitge Biomedical Investigation Institute), Barcelona, Spain

**Keywords:** Acellular dermal matrix, Perianal fistula, Complex fistula, ADM, Plug

## Abstract

**Backgound:**

Effective, standardized treatments for complex anal fistula (CAF) still represent a clinical challenge. Emerging procedures attempted to achieve the healing rates of fistulotomy whilst preserving sphincter function. Acellular dermal matrix (ADM) used as a plug inserted through the fistulous tract is among newer treatment options. Varying success rates have been reported, most with short-term follow-up. The aim of this study was to report the long-term results of ADM-plug for CAF.

**Methods:**

Retrospective analysis of a prospective database of patients treated with CAF. All consecutive patients presenting at two tertiary centers (Vall d’Hebron University Hospital and Bellvitge University Hospital, Barcelona, Spain) between November 2015 and March 2019 with a single, cryptoglandular CAF were evaluated for treatment with an ADM-plug were included. The primary endpoint was absence of discharge at clinical examination at 12 month follow-up.

**Results:**

Twenty-two patients were included [7 women and 15 men, median age 56 (33–74) years]. Most patients had high transsphincteric fistulas (63.6%). The median follow-up was 42 (21–53) months. The 12 month success rate was 68.2%, with an overall healing rate of 59.1%. 77.8% of recurrences occurred within 12 months from surgery. One plug extrusion was observed. No major complications or mortality occurred during the follow-up. Patients did not report any worsening of fecal continence.

**Conclusions:**

This pilot study showed that more than half of patients with CAF could benefit from ADM-plug placement, preserving continence. A minimum follow-up of 12 months is recommended, because most recurrences occur during the first year.

**Supplementary Information:**

The online version contains supplementary material available at 10.1007/s10151-022-02593-1.

## Introduction

Anal fistula treatment depends on fistula anatomy, the cause of the fistula and the amount of involved external anal sphincter muscle [1, 2]. Surgery for complex anal fistulas (CAF) is associated with higher recurrence rates and higher risk of injury to the anal sphincter complex, resulting in flatus and/or fecal incontinence [3, 4]. The risk of incontinence is higher in women and in anteriorly located fistulas [4–6].

Surgery for CAF often involves staged procedures, with seton placement and delayed definitive treatment. Key steps to success include control/elimination of acute sepsis and secondary extensions and adequate removal of any chronic granulation or epithelial tissue lining the fistula [4]. Fistulotomy achieves the highest healing rates, but it implies a potential risk of continence worsening or “de novo” incontinence, reported in more than 10% of patients [7]. Advancement flaps represent a viable option for CAF, with success rates ranging from 40 to 80% [7–11]. Incontinence may also be a complication of the latter procedure, being as high as 20% [7].

To avoid or diminish postoperative incontinence, a wide range of techniques and technologies have been proposed, i.e., ligation of intersphincteric fistula tract (LIFT), fibrin glue injection, non-dermis based anal fistula plug, video-assisted anal fistula treatment (VAAFT) and fistula laser closure (FiLaC). The aim of these treatments is to achieve the highest healing rates, whilst preserving sphincter function. Different success rates have been reported, ranging from 12.5 to 88% [5, 8, 9, 12–17]. However, few studies report the long-term effectiveness of the procedures, and results have not been consistently replicated.

Development and availability of biocompatible materials offer a new treatment option for CAF, including a plug of acellular dermal matrix (ADM). This treatment respects the anatomy and physiology of the anal canal and can be repeated if needed. However, long-term data on efficacy and safety are necessary.

The aim of this study was to report the long-term (minimum of 12 months) fistula healing rates of an ADM-plug for CAF. Secondary aims included detailing its adverse events.

## Materials and methods

This was a retrospective analysis of a prospectively maintained database of patients with CAF treated at two tertiary centers (Vall d’Hebron University Hospital and Bellvitge University Hospital, Barcelona, Spain). All patients provided written informed consent to receive treatment. The study was conducted following the Strengthening the Reporting of Observational Studies in Epidemiology (STROBE) Statement [18].

Data from all consecutive patients presenting with CAF who underwent surgery between November 2015 and March 2019 were evaluated for inclusion in the analysis.

Patients underwent surgery with a porcine ADM-plug placement (PressFit^®^, Decomed distributed by Biocablan), and were subsequently followed-up.

### Inclusion and exclusion criteria

All consecutive patients with a single, cryptoglandular CAF were included in the analysis. Patients with multiple fistulas, Crohn’s disease and simple fistulas were excluded from the study.

### Definitions

Fistulas were defined CAF according to Park’s classification [19].

Clinical recurrence was defined as the presence, at clinical examination, of any perianal suppuration at follow-up > 6 months. In case of equivocal findings, endoanal ultrasonography (EAUS) and/or magnetic resonance imaging (MRI) were performed.

Disease persistence was defined as ongoing symptoms of discharge since surgery. Both recurrence and persistence were considered treatment failure or no healing.

Continence impairment was defined as any new symptoms that were not present before the procedure. This was recorded at all follow-up appointments.

### Endpoint and outcome measures

The primary endpoint was clinical fistula healing (absence of suppuration on gentle compression of perianal region) at 12 month follow-up.

Secondary endpoints included overall success, adverse events, plug-specific issues, factors associated with recurrence or disease persistence, and clinically relevant problems with continence.

### Perioperative management

Prior to ADM-plug treatment, all patients received an examination under anesthesia (EUA) to accurately define the anatomy of the fistula, along with loose seton placement to control sepsis. Definitive surgery was subsequently performed after a minimum of 8 weeks.

All surgical procedures were carried out as day-case interventions by colorectal surgeons with expertise in proctology and perianal fistula treatment.

### Surgical technique

All patients receive two fleet enemas prior to the surgery. The procedure is performed under spinal anesthesia. Those patients with an anterior fistula are operated on in prone position (Jack knife position), whilst lithotomy is used for posteriorly located fistulas. Aqueous chlorhexidine is used for skin antisepsis (iodine is not recommended for this procedure).

**Fig. 1:**
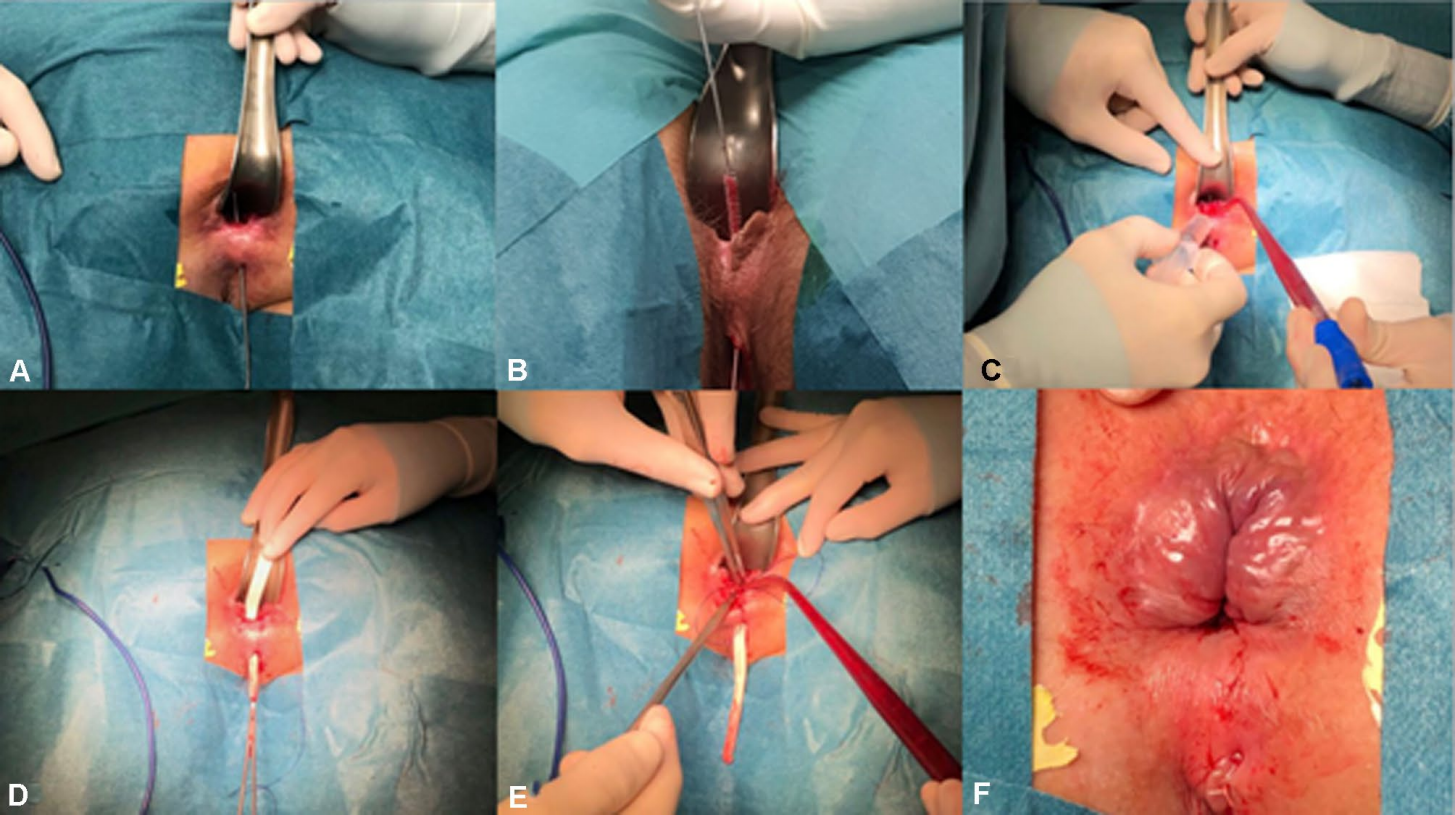
Patient in prone position. **A** Overview of fistula tract. **B** Curettage removing scarring tissue. **C** Cleaning the tract with saline solution. **D** ADM-plug into the tract with the wide part in the internal orifice. **E** Stitching the plug to the internal orifice. **F** Plug stitched on opened external orifice. *ADM* acellular dermal matrix

Once indications for plug placement are confirmed, the ADM-plug is soaked in saline for 20 min. After removing the seton and after adequate curettage of the fistula track, the latter is irrigated with saline. Oxygen peroxide should not be used. The ADM-plug is inserted along the fistula tract, positioning the wide part of the plug at the internal opening, where it is transfixed with an absorbable 2/0 suture, which closes the opening. A small mucosal “pocket” can also be used to cover the internal opening and the plug, as an alternative to direct stitching. This pocket is created with circular resection of the internal opening and radial dissection of the mucosa and submucosa from internal sphincter. It is closed with interrupted stitches covering the plug with healthy tissue. The excess plug is cut at the level of the external opening, and a stitch is placed to fix it, making sure to leave the external orifice open to allow drainage (Fig. 1).

Patients are discharged the day of surgery, with oral analgesia and laxatives.

### Statistical analysis

Continuous variables are reported as medians with ranges. Categorical values are reported as absolute numbers with percentages.

## Results

**Fig. 2:**
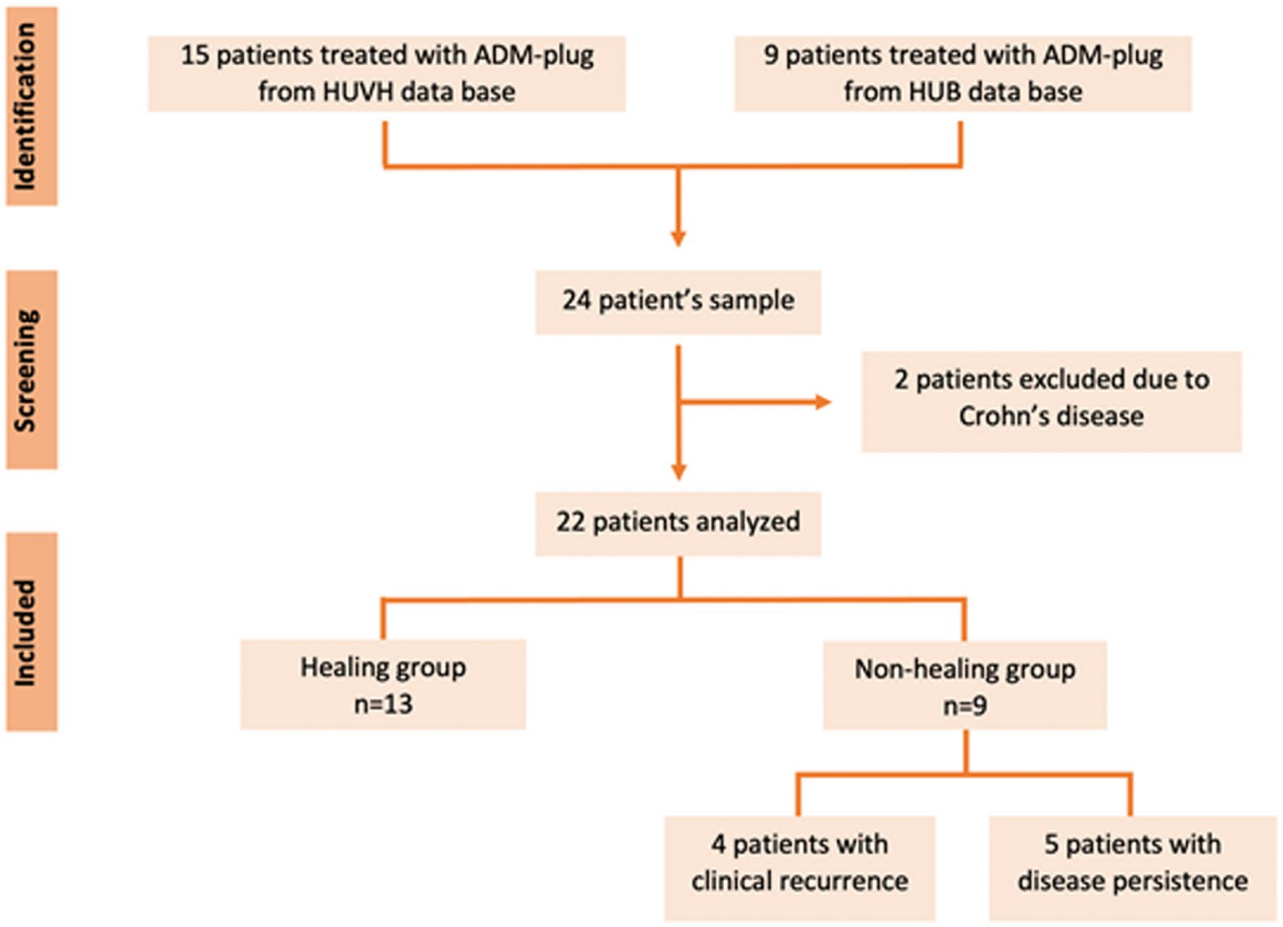
Flowchart of patients included in the study. *ADM* acellular dermal matrix, *HUB* Hospital Universitario de Bellvitge, *HUVH* Hospital Universitario Vall d’Hebron

During the study timeframe, 24 consecutive patients with single CAF were treated with an absorbable porcine ADM pyramidal (110 × 11 × 4.5 mm) plug (PressFit^®^) and had a minimum of 12 months of follow-up. Two patients were excluded from the analysis, because they were diagnosed with Crohn’s disease (Fig. 2).

Twenty-two patients with CAF, 7 (31.8%) women and 15 (68.2%) men, were included. The median age was 56 (33–74) years. According to Parks’ classification, 68.2% of patients had suprasphincteric or high transsphincteric fistulas, and 31.8% had middle or low transsphincteric fistulas; all of them with a single track. The fistulas were anterior in 9 (41%) patients and posterior in 13 (59%). The baseline patient characteristics are summarized in Table 1*.*

**Table 1:** Baseline patient characteristics

Variable	
Age, median (range), years	55 (33–74)
Sex, *n* (%)	7 F (31.8%)–15 M (68.2%)
Ulcerative colitis (cryptoglandular)	3 (13.6%)
Immunosuppression, *n* (%)	2 (9.1%)
Park’s classification	
Supra/transsphincteric fistula	68.2%
Middle/low transsphincteric fistula	31.8%
Fistula location	
Anterior	41%
Posterior	59%
Previous procedures for CAF	
1st treatment	36.4%
2nd line	31.8%
3rd line	27.3%
4th line	9.1%

*CAF*: complex anal fistula

Fourteen patients (63.6%) had previously had more than one surgical procedure: sequential fistulotomy (*n* = 12), placement of a synthetic polyglycolic acid trimethylene carbonate plug (*n* = 2), LIFT procedure (*n* = 3), over the scope clip (OTSC; *n* = 2), endoanal advancement flap (*n* = 2), and collagen paste placement (*n* = 2). The median time between last treatment and ADM-plug surgery was 17 (2–48) months. The median time between placement seton placement and definitive surgery was 15.8 (1–42) months, with a non-significant difference between the healing group (11.5 months) and the non-healing group (22.1 months). Median operative time was 48 (23–100) minutes. A mucosal pocket was used in 17 patients. No intraoperative adverse events were recorded.

### Primary aim: fistula healing

The median length of follow-up was 42 (21–53) months.

The overall success rate of ADM treatment was 59.1%. There were similar results in recurrence rate between first-line treatment from those who were recurrent from other treatments (37.5% vs 42.9%). The median time to recurrence was 9 (3–18) months, with 77.8% of patients having a recurrence within 12 months after surgery (Fig. 1). Failure occurred in nine patients, five who never improved (disease persistence) and four who had improvement and then had a recurrence.

### Secondary aims

No morbidity or mortality occurred during the follow-up. No change in fecal continence was reported. One patient had a plug extrusion, which required replacement 3 months after the primary procedure. This patient had initial resolution of symptom, with no anal suppuration. Months later, a recurrence was observed during follow-up at an outpatient visit as suppuration from the previous fistula orifice was noted.

The cohort was divided into two groups, according to the outcome: *healing* vs *no healing*. The latter included disease persistence and recurrence. Comparisons of baseline and fistula characteristics are reported in Tables 2 and 3.

**Table 2:** Comparison of baseline characteristics according to the outcome

Variable	Healing	No healing/recurrence	*p* value
Age, median (range) years	58 (44–74)	46 (33–74)	0.0823
Sex	2F–11M	5 F–4M	0.074
Smoking	4 (30.8%)	4 (44.4%)	0.662
Obesity (BMI ≥ 30 kg/m^2^)	2 (15.4%)	1 (11.1%)	> 0.99
Immunosuppression	1 (11.1%)	1 (11.1%)	> 0.99
Diabetes mellitus	3 (23.1%)	1 (11.1%)	0.616
Arterial hypertension	6 (46.1%)	1 (11.1%)	0.07

*BMI* body mass index

**Table 3:** Comparison of fistula characteristics according to the outcome

Fistula characteristics	Healing group	Non-healing group	*p *value
Time between seton placement and surgery—median (range), months	12.6 (2–37)	23.2 (7–48)	0.18
Type of fistula			0.65
Suprasphinteric and high transsphincteric—*n* (%)	8 (53.3%)	7 (46.7%)	
Middle and low transsphincteric—*n* (%)	5 (71.4%)	2 (28.6%)	
Location			> 0.99
Anterior—*n* (%)	5 (55.6%)	4 (44.4%)	
Posterior—*n* (%)	8 (61.5%)	5 (38.5%)	
Mucosal pocket added			> 0.99
Mucosal pocket—*n* (%)	10 (58.8%)	7 (41.2%)	
No mucosal pocket—*n* (%)	3 (60%)	2 (40%)	
Line of treatment			> 0.99
First-line treatment—*n* (%)	5 (62.5%)	3 (37.5%)	
Recurrent fistula—*n* (%)	8 (57.1%)	6 (42.9%)	

Regarding the patients in whom the treatment with ADM-plug failed (*n* = 9), four had a loose seton in place at 36 month follow-up and declined any additional procedures, due to a stabilization of their symptoms. Another patient still had some discharge at last available follow-up but refused any treatment (including seton placement), because of the improvement of the baseline status. Two patients were treated with Permacol paste, which was successful in one; the other one declined any additional procedure because of improved perceived quality of life.

Another patient was treated with platelet-rich plasma (PRP) and reported an absence of symptoms at 4 month follow-up.

The last patient was treated with a partial-thickness advancement flap, with no recurrence at 4 year follow-up. However, the patient reported soiling and urgency. Data are summarized in Table 4.

**Table 4:** Secondary treatments and outcomes

Secondary treatment	No more surgery	Seton placement	Advancement flap	PRP	Permacol paste
Patients, *n* (%)	2 (22.2%)	3 (33.3%)	1 (11.1%)	1 (11.1%)	2 (22.2%)
Healing, *n* (%)	0 (0%)	0 (0%)	1 (100%)	1 (100%)	1 (50%)
No healing, *n* (%) (increased well-being)	2 (100%)	3 (100%)	–	–	1 (50%)

*PRP* platelet-rich plasma

## Discussion

This is the first study to assess the long-term outcomes after treatment of CAF with an ADM-plug. Most studies published to date are based on a different ADM-plug, described as non-crosslinked, collagen-based extracellular matrix (ECM) material derived from porcine small intestinal submucosa (Fistula Biodesign plug—Cook Surgisis^®^). The results reported in those studies for perianal fistula healing rates range from 12.5 to 88% [5, 8, 9, 12–17]. There can be various explanations for such wide variations in healing rates, mainly consisting of different definitions for “healing”, type of fistulas included, and duration of follow-up (ranging from 1 week to 18 months). The extrusion rates reported with previous plugs are between 1.6 and 20% [5, 6, 8, 9, 12] compared to 4.5% with the ADM-plug used in the current study.

**Fig. 3:**
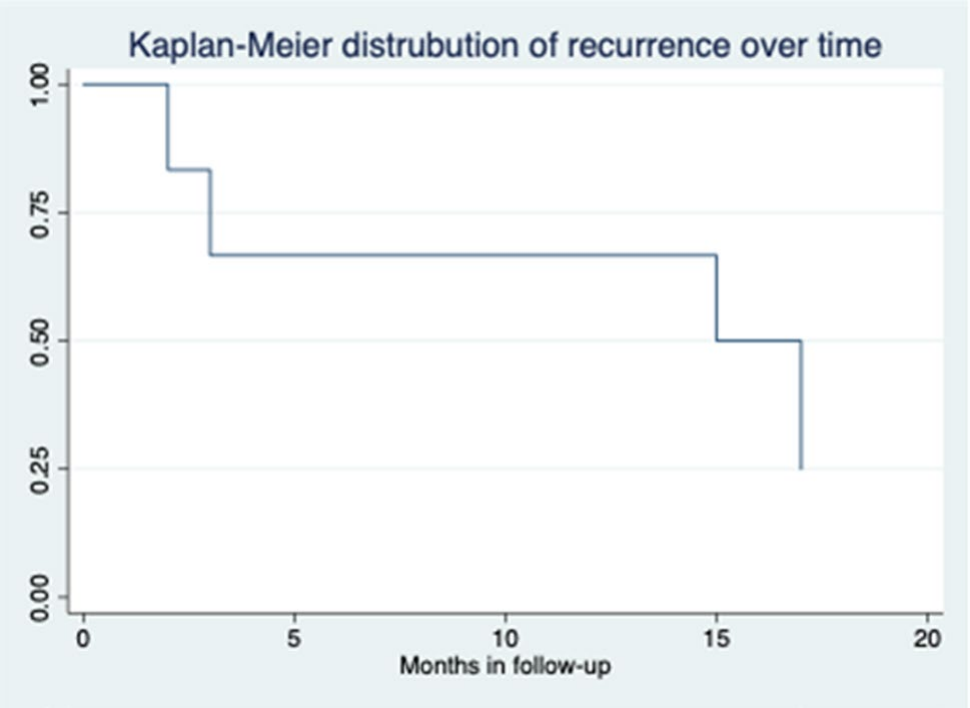
Overall distribution of recurrence over time (40.9% “no healing”)

In this study, the overall success rate of ADM-plug treatment was 59.1% at a median follow-up of 42 months. Unsurprisingly, recurrence rates increase with longer follow-up (Fig. 3). All recurrences in this study occurred within 18 months after treatment. Therefore, an adequate follow-up period is recommended to have a more realistic recurrence rate. This should be at least 12 months, by when more than 75% recurrences were observed in this series. Inadequate follow-up duration is among the factors that contribute to the “honey moon” observed with newer technologies or techniques for CAF treatment, i.e., the excellent results that are commonly observed in pilot reports; which can be later confuted in studies with appropriate follow-up duration and modality.

Interestingly, five out of nine patients in the no healing group, four of whom had seton placement after the fistula recurrence, also had an improvement of their baseline status, with higher perceived quality of life than before surgery, so that they declined another surgical treatment.

Due to the small sample size, no definitive conclusions can be drawn concerning patient and fistula characteristics that can predict failure. Middle and low transsphinteric fistulas seemed to have a greater chance of healing (71.4% vs 53.3% with suprasphinteric and high transsphinteric fistulas), however, this did not reach statistical significance.

This study has some limitations. It was a retrospective analysis, with no control groups. Another limitation is that mucosal flaps to cover the internal opening were used in most patients in the current series (77.3%), potentially impacting the outcome. Balciscueta et al. in their systematic review and meta-analysis, reported 69.9 (60–80)% healing rates for CAF treated with a mucosal advancement flap alone, with follow-up ranging from 3 to 71 months [7]. Although the mucosal pocket is not a proper advancement flap, this factor can lead to overestimate the success of the ADM-plug due to the benefit of two different techniques in one procedure. Of note, there was no difference in healing rate comparing the mucosal pocket group with the group of patients who only received suture of the internal opening (58.8% vs. 60%). On the other hand, the reported incontinence rates after mucosal advancement flap range between 0 and 14.7% [7], higher than those reported by Han et al. with ADM-plugs (1.75%) [6].

Another factor that makes it difficult to compare the current study with previous series is the variability of operative techniques and perioperative care between studies. In our series, shorter intervals between seton placement and surgery are associated with higher rates of healing (12.6 months in the *healing* group vs 23.2 months in the *no healing* group).

Costs are a major drawback of the ADM-plug. This treatment is a more expensive option than classical techniques which do not require special materials or devices, like fistulotomy, fistulectomy, advancement flaps, or LIFT. Nevertheless, the fact that ADM-plug is not associated with short- or long-term morbidity, along with the improvement in patient well-being (irrespective of CAF healing), suggest that such treatment might be of value in the long term, potentially reducing secondary costs.

## Conclusions

This pilot study showed that more than half of the patients with CAF could benefit from ADM-plug placement, with no effect on continence. This technique can be offered as a first-line treatment of CAF, especially in those patients with some previous degree of incontinence. Shorter waiting time between seton placement and definitive surgery is associated with higher healing rates.

A minimum follow-up of 12 months is desirable to reliably assess the outcome of such treatment in future studies, ideally reaching 18 months.

The small sample size in this study, as well as the use of a mucosal pocket to cover the plug, make it difficult to draw definitive conclusions, but the promising results achieved in terms of continence preservation and well-being improvement warrant further exploration in randomized controlled trials, provided that adequate follow-up is planned.

## Supplementary Information

Below is the link to the electronic supplementary material.

Supplementary file1 (DOC 86 KB)
